# Characterizing antimicrobial activity of environmental *Streptomyces* spp. and oral bacterial and fungal isolates from *Canis familiaris* and *Felis catus*

**DOI:** 10.1128/msphere.00098-25

**Published:** 2025-04-14

**Authors:** Audrey Cowen, Bonnie Yiu, Sara Fallah, Kirsten J. Meyer, Emily Puumala, Yunjin Lee, Haley L. Zubyk, Nicole Robbins, Justin R. Nodwell, Jessie MacAlpine, Leah E. Cowen

**Affiliations:** 1 Department of Molecular Genetics, University of Toronto 204248, Toronto, Ontario, Canada; 2 Department of Biochemistry, Temerty Faculty of Medicine, University of Toronto 233836, Toronto, Ontario, Canada; 3 David Braley Centre for Antibiotic Discovery, M. G. DeGroote Institute for Infectious Disease Research, Department of Biochemistry and Biomedical Sciences, McMaster University 536887, Hamilton, Ontario, Canada; 4 Department of Cell and Systems Biology, University of Toronto 98684, Toronto, Ontario, Canada; University of Georgia, Athens, Georgia, USA

**Keywords:** *Candida albicans*, *Escherichia coli*, Streptomyces, *Canis familiaris*, *Felis catus*, antimicrobials, resistance, drug combinations

## Abstract

Antimicrobials are a pillar of modern medicine, yet our limited arsenal of antibiotics and antifungals is currently threatened by widespread drug resistance. Ongoing efforts are focused on developing strategies to identify compounds that enhance the efficacy of current antimicrobials and develop novel, resistance-evasive therapeutic strategies. In this study, we characterized microbial isolates from two distinct environments to identify those that exhibit antimicrobial activity alone and in combination with current antimicrobials: (i) oral isolates from domesticated animals and (ii) environmental *Streptomyces* spp. First, conditioned media prepared from bacterial and fungal oral isolates that were collected from *Canis familiaris* and *Felis catus* were screened for antibacterial and antifungal activity. Three supernatants from bacterial isolates exhibited antifungal activity against the human fungal pathogen *Candida albicans* in the presence of subinhibitory concentrations of fluconazole, the most widely deployed antifungal. Additionally, two bacterial isolates displayed antibacterial activity against *Escherichia coli* alone and in combination with the antibacterial ampicillin. Furthermore, 32 environmental isolates of confirmed and predicted *Streptomyces* spp. were screened for activity against *C. albicans* and *E. coli*. Cell-free media harvested from isolates WAC5038 and WAC5287 exhibited antifungal activity against *Candida* spp., while only the WAC5038-conditioned medium displayed antibacterial activity. Bioactivity-guided fractionation, coupled with UV/Vis absorbance spectra, suggested that the bioactive compound in WAC5287 has a similar absorbance spectrum to the antifungal class of polyenes, while the bioactive component of WAC5038 remains unknown. Overall, this work highlights a strategy to collect and screen environmental isolates for the identification of novel antimicrobials.

**IMPORTANCE:**

The emergence and spread of antimicrobial resistance presents a global health challenge. As such, researchers are focused on developing pipelines to discover novel antimicrobials. In this study, we screened two distinct collections of microbes for antimicrobial activity. First, we collected bacterial and fungal isolates from the oral cavities of domesticated dogs and cats and identified these isolates using 16S (bacteria) and ITS (fungi) sequencing. Follow-up analyses confirmed that some conditioned media from bacterial isolates had antibacterial activity against *Escherichia coli* and antifungal activity against *Candida albicans* both alone and in combination with the current antimicrobial drugs. Additionally, screening 32 predicted or confirmed Streptomyces environmental isolates for antifungal and antibacterial activity identified two isolates with antifungal activity (WAC5038 and WAC5287), with only one isolate demonstrating antibacterial activity (WAC5038). Overall, this study provides a framework to identify and characterize environmental microbes with antimicrobial activity.

## OBSERVATION

Antimicrobials have revolutionized modern medicine; however, the rise of antimicrobial resistance has resulted in a dire need to accelerate antibiotic and antifungal discovery (1–3). Many classes of antimicrobials are of natural product origin, including the antifungal polyenes and the antibacterial beta-lactams (4). Current research is focused on developing pipelines to discover novel compounds with activity against diverse pathogens and leveraging combination therapy to hinder drug resistance and restore antimicrobial efficacy (5–7). Here, we aimed to identify the antimicrobial activity of microbes from two environmental niches: (i) oral isolates from *Canis familiaris* and *Felis catus* and (ii) environmental *Streptomyces* spp.

**Fig 1:**
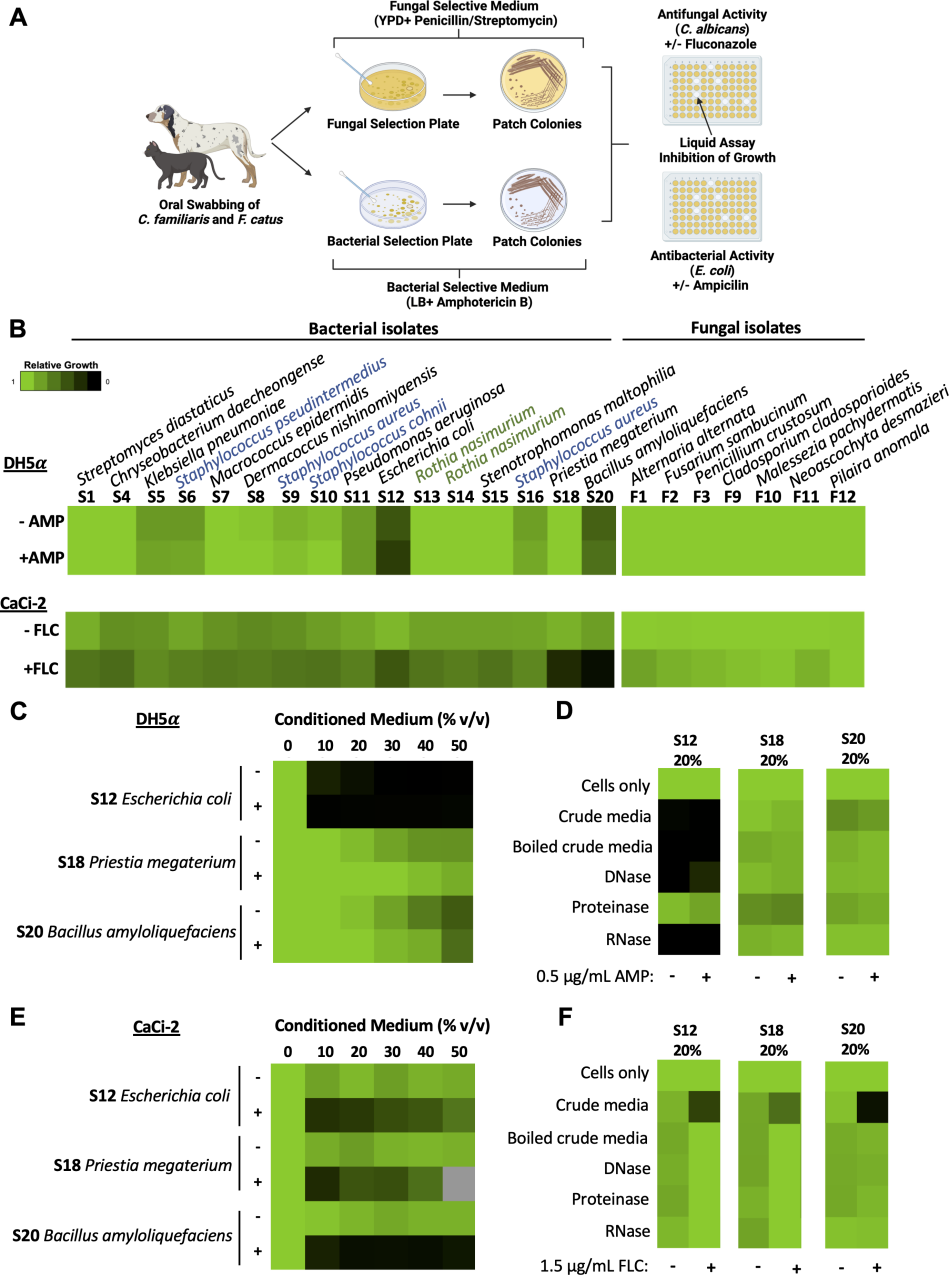
Isolates from *F. catus* and *C. familiaris* exhibit antimicrobial activity. (A) Schematic depicting the workflow of swabbing *C. familiaris* and *F. catus* for microbial samples and assessing for antimicrobial activity. Schematic created in BioRender. (**B**) Growth inhibitory assays were conducted using 50% (vol/vol) conditioned media in a 96-well plate with 200 µL total volume per well. Bacterial inoculum was diluted to an OD_600_ of 0.01 in LB, and 100 µL of inoculum was added to 100 µL conditioned medium per well in a 96-well plate, in the absence and presence of subinhibitory concentrations of ampicillin (0.5 µg/mL). Relative growth was measured by absorbance at 600 nm (OD_600_) after a 24-hour incubation at 37°C (see color bar). Fungal inoculum was diluted to 1 × 10^4^ cells/mL in YPD, and 100 µL of inoculum was added to 100 µL conditioned medium per well in a 96-well plate, in the absence and presence of subinhibitory concentrations of fluconazole (1.5 µg/mL). Relative growth was measured by absorbance at 600 nm (OD_600_) after 48 hours at 30°C (see color bar). Microbial isolates belonging to the same genera are color-coded when multiple species were recovered from that genus. Blue: *Staphylococcus*. Green: *Rothia*. (**C**) Dose-response assays were conducted using a titration of 10, 20, 30, 40, or 50% (vl/vol) conditioned media in LB alone or in combination with subinhibitory concentrations of ampicillin (0.5 µg/mL). Experiments were conducted as described in panel **B**. (**D**) Conditioned media were incubated at 37°C for 1  hour with 100  mg/L DNase I, RNase A, or proteinase K. Enzymes were inactivated by heating the treated conditioned media at 100°C for 10  minutes. The activity of the conditioned media (20% vol/vol) was assessed against DH5α as described in panel **B**. (**E**) Dose-response assays were conducted as described in panel **B,** without or with subinhibitory concentrations of fluconazole (1.5 µg/mL). Grey box: bioactivity could not be determined due to conditioned media precipitating out of solution. (**F**C) Conditioned medium was incubated at 37°C for 1  hour with 100  mg/L DNase I, RNase A, or proteinase K. The activity of the conditioned media was assessed by incubating CaCi-2 at 30°C for 48  hours in the presence (+) or absence (−) of 1.5 µg/mL of fluconazole. Fungal growth was quantified by measuring OD_600_. All data represent technical duplicates in two biological replicates.

Antimicrobial activity was predicted to be present in the oral mucosa of domestic *C. familiaris* and *F. catus* due to their lifestyle and diet resulting in frequent contact with potentially pathogenic organisms, as well as research that has identified microbes from diverse anatomical sites in animals and other living creatures (8–10). To determine if microbes from the oral mucosa of domestic mammals had antimicrobial activity, volunteers were recruited to collect microbial samples from the oral cavity of *C. familiaris* and *F. catus* domestic animals. Sterile swabs from 20 animals were dipped in PBS and rubbed along the gums, tongue, cheeks, and teeth of each animal in a non-invasive manner (Fig. 1A). For each animal, two swabs were collected: one was plated on fungal-selective medium (Yeast Peptone Dextrose [YPD] supplemented with 100 µg/mL penicillin and 100 µg/mL streptomycin), and the other was plated on bacterial-selective medium (Luria-Bertani [LB] broth supplemented with 4 µg/mL amphotericin B). Plates were incubated at 37°C for 24 hours for bacterial growth or at 30°C for 5 days for fungal growth. Of the hundreds of colonies observed on the selection plates, phenotypically distinct fungal and bacterial colonies were selected from each animal and were propagated onto fresh selection plates. From there, colonies were selected for 16S and ITS sequencing from each animal to identify the bacterial and fungal species, respectively, revealing a diverse collection of species in the oral cavities of domesticated animals (Table 1).

**TABLE 1:** 16S and ITS sequencing results from samples collected from *C. familiaris* and *F. catus*

Isolate type	Sample	Origin of the sample	Species	% identity
Bacterial isolates	S1	*C. familiaris*	*Streptomyces diastaticus*	99.47
	S4	*C. familiaris*	*Chryseobacterium daecheongense*	100
	S5	*F. catus*	*Klebsiella pneumoniae*	100
	S6	*C. familiaris*	*Staphylococcus pseudintermedius*	98.68
	S7	*C. familiaris*	*Macrococcus epidermidis*	98.42
	S8	*F. catus*	*Dermacoccus nishinomiyaensis*	98.12
	S9	*F. catus*	*Staphylococcus aureus*	98.71
	S10	*C. familiaris*	*Staphylococcus cohnii*	100
	S11	*C. familiaris*	*Pseudomonas aeruginosa*	100
	S12	*C. familiaris*	*Escherichia coli*	100
	S13	*C. familiaris*	*Rothia nasimurium*	99.58
	S14	*C. familiaris*	*Rothia nasimurium*	99.55
	S15	*F. catus*	*Stenotrophomonas maltophilia*	100
	S16	*C. familiaris*	*Staphylococcus aureus*	100
	S18	*F. catus*	*Priestia megaterium*	96.09
	S20	*C. familiaris*	*Bacillus amyloliquefaciens*	100
Fungal isolates	F1	*C. familiaris*	*Alternaria alternata*	100
	F2	*C. familiaris*	*Fusarium sambucinum*	100
	F3	*C. familiaris*	*Penicillium crustosum*	100
	F9	*F. catus*	*Cladosporium pseudocladosporioides*	100
	F10	*C. familiaris*	*Malassezia pachydermatis*	99.63
	F11	*C. familiaris*	*Neoascochyta desmazieri*	100
	F12	*C. familiaris*	*Pilaira anomala*	99.66

To probe for antifungal or antibacterial activity in the *F. catus* and *C. familiaris* isolates, conditioned media from a single isolate, prioritized based on defined 16S/ITS sequencing and phenotypically diverse colony morphology, was made by growing strains overnight in either YPD at 30°C (for fungal isolates) or LB at 37°C (for bacterial isolates). The following day, cells were pelleted at 16,000  ×  *g* for 5  minutes, and the resulting supernatant was passed through a 0.2 µm filter. Antifungal activity was assessed against an azole-tolerant clinical isolate of the human fungal pathogen, *Candida albicans* (CaCi-2) (11), in the absence and presence of a subinhibitory concentration of the antifungal fluconazole (1.5 µg/mL). Antibacterial activity was assessed against the Gram-negative bacterium, *Escherichia coli* (DH5α), in the absence and presence of a subinhibitory concentration of ampicillin (0.5 µg/mL). *C. albicans* was grown overnight at 30°C in YPD to saturation and diluted to 1 × 10^4^ cells/mL in YPD. *E. coli* was grown overnight at 37°C in LB and diluted to an OD_600_ of 0.01 in LB. Equal parts inoculum and conditioned medium were added to each well of a 96-well plate. Plates were incubated at 30°C for 48 hours or 37°C for 24 hours to assess fungal or bacterial growth, respectively.

Conditioned media from two of the bacterial isolates, namely, *E. coli* (S12) and *Bacillus amyloliquefaciens* (S20), displayed bioactivity against *E. coli* in the absence and presence of a subinhibitory concentration of ampicillin (Fig. 1B). In addition, conditioned media from three bacterial isolates, namely, *E. coli* (S12), *Priestia megaterium* (S18), and *Bacillus amyloliquefaciens* (S20) had inhibitory activity against *C. albicans* in the presence of a subinhibitory concentration of fluconazole. Next, to evaluate the potency of prioritized conditioned media, a titration of each sample was performed against *E. coli* (Fig. 1C) and *C. albicans* (Fig. 1E). Conditioned medium S12 displayed antibacterial activity alone and in combination with ampicillin, with activity observed in as low as 10% (vol/vol) (Fig. 1C). Conditioned medium S20 displayed moderate bioactivity against *E. coli*, reducing growth by ~45% at the highest concentration of 50% (vol/vol) relative to no treatment. Against *C. albicans*, all prioritized conditioned media from bacterial isolates (S12, S18, and S20) displayed antifungal activity as low as 10% (vol/vol) in combination with fluconazole.

Finally, to explore whether the activity in the conditioned medium was due to secreted DNA, RNA, or protein, the cell-free supernatant was treated with DNase I, RNase A, or proteinase K, as previously described (12). Against *E. coli*, S12 lost bioactivity only after proteinase K treatment, suggesting that the bioactive component is a protein. Boiling of crude S20-conditioned medium alone, a process conducted as a control given that heat was used to inactivate the enzymes, resulted in the loss of antibacterial activity (Fig. 1D). Similarly, boiling S12, S18, and S20 also resulted in the loss of antifungal activity in the presence of fluconazole, suggesting that the component responsible for antimicrobial activity is susceptible to heat and/or there are multiple components present in the supernatant responsible for activity (Fig. 1F).

**Fig 2:**
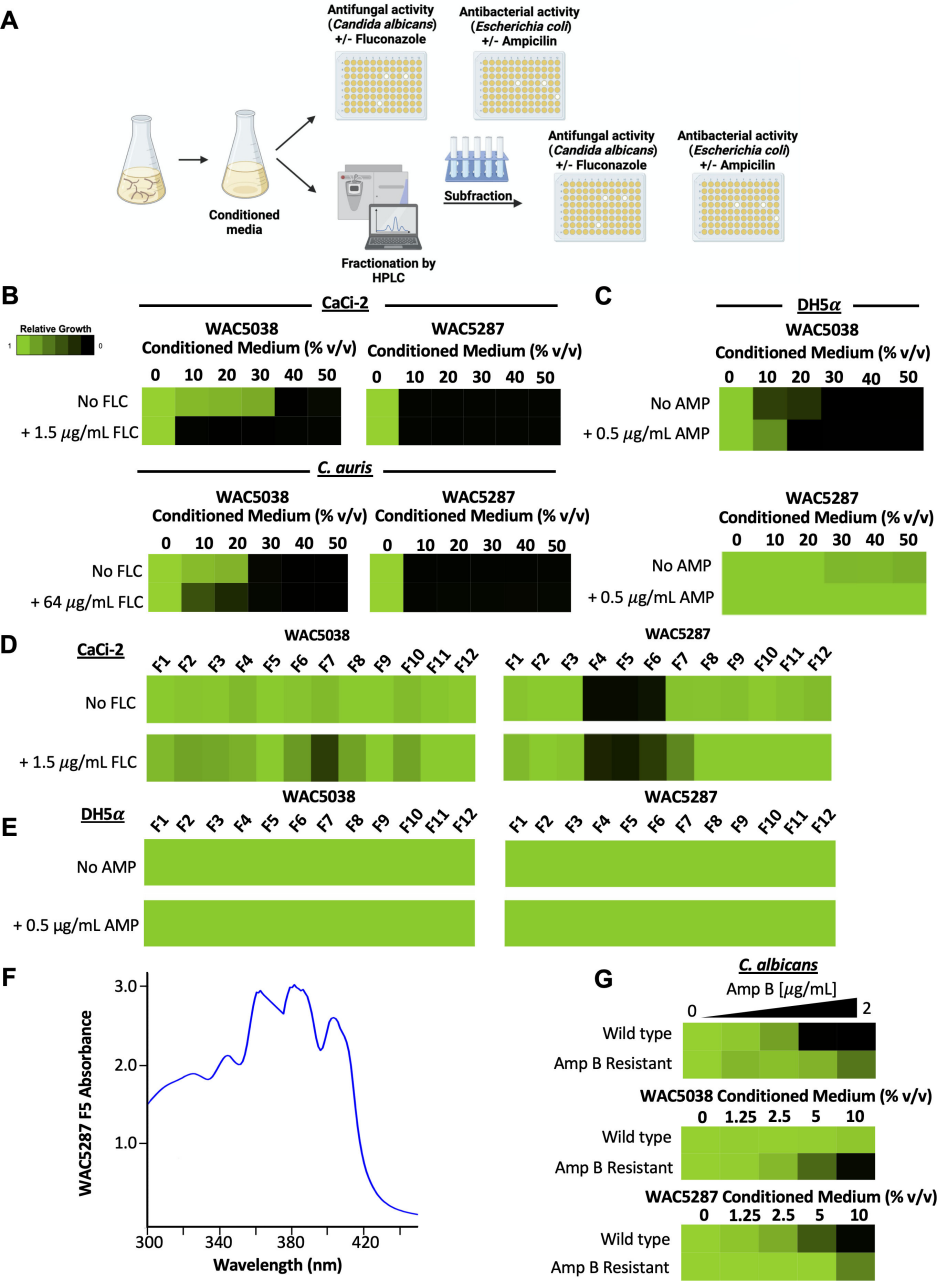
Conditioned media from distinct *Streptomyces* spp. have antimicrobial activities. (**A**) Schematic depicting the workflow for testing conditioned media from *Streptomyces* spp. for bioactivity. Schematic created in BioRender. (**B**) Conditioned media from two prioritized *Streptomyces* spp., WAC5038 and WAC5287, show bioactivity against *C. albicans* (CaCi-2) and *C. auris* (Ci6684). Twelve subfractions from conditioned media (10 mL) were generated by butanol extraction followed by separation by HPLC, eluting from a C18 column with a step gradient from 5% to 97% acetonitrile with 0.1% formic acid. Dose-response assays were conducted as described in Fig. 1C against *C. albicans* or *C. auris* in the presence or absence of fluconazole (see color bar). (**C**) Dose-response assay with subfractions from two prioritized *Streptomyces* spp., WAC5038 and WAC5287, against *E. coli* (DH5ɑ) were performed, as described in Fig. 1C. WAC5038 displayed antibacterial activity as low as 10% (vol/vol), with a slight decrease in bioactivity observed with the addition of ampicillin. Conditioned medium from WAC5287 displayed no antibacterial activity. (**D**) Subfractions of butanol-extracted conditioned media were generated by HPLC. Dried subfractions were dissolved in 20 µL of DMSO. *C. albicans* cells (1 × 10^3^) in YPD were seeded per well in a 96-well plate (200 µL total volume), and 2 µL of each subfraction was added. Relative growth was measured by OD_600_ after a 48-hour incubation at 30°C. (**E**) Subfractions were tested as described above. *E. coli* inoculum was diluted to an OD_600_ of 0.01 before being adding to the 96-well plate. Relative growth was measured by OD_600_ after a 24-hour incubation at 37°C. (**F**) Absorbance spectra of WAC5287 fraction 5 (rt 6.0 min) reveal the triple absorption peaks (at 362, 382, and 482 nm) highly characteristic of a polyene. (**G**) *C. albicans* amphotericin B-resistant isolate is resistant to WAC5287 conditioned medium and is hypersensitive to WAC5038 conditioned medium. Dose-response assays were conducted, as described in Fig. 1C, and incubated for 24 hours at 30°C. All dose-response assays were conducted in technical duplicates and are representative of biological replicates.

Previous work demonstrated the broad antimicrobial potential of *Streptomyces* spp., including the production of the common antibacterial streptomycin and the polyene class of antifungals (2, 13). To screen for antimicrobial agents from *Streptomyces* spp., a collection of 32 environmental isolates from diverse geographic sites was evaluated for their antifungal and antibacterial activity using agar plate assays (14) (data not shown). Through this approach, we identified that *Streptomyces sampsonii* (WAC5287) and a predicted *Streptomyces* sp. (WAC5038) demonstrated antibacterial and antifungal activity. To assess whether the activity persisted in cell-free *Streptomyces* conditioned media, *Streptomyces* spp. were grown for 7 days at 30°C in Bennett’s medium, and conditioned media were prepared as previously described (12) (Fig. 2A). Antifungal activity was evaluated in a liquid dose-response assay against *C. albicans* (CaCi-2) (11) and the emerging fungal pathogen *Candida auris* (Ci6684) (15, 16), with or without fluconazole, as described above (Fig. 2A). The conditioned media from WAC5287 and WAC5038 were both able to inhibit the growth of *C. albicans* and *C. auris* alone. Furthermore, the conditioned medium from WAC5038 was enhanced by a subinhibitory concentration of fluconazole (Fig. 2B). Enhanced activity was not observed with WAC5287 in the presence of fluconazole, as the lowest concentration tested displayed potent single-agent activity. Interestingly, only conditioned media from WAC5038 displayed antibacterial activity against *E. coli*, where the addition of ampicillin caused minimal change in bioactivity (Fig. 2C). To characterize the bioactive component(s) of WAC5038 and WAC5287, we fractionated conditioned media using high-performance liquid chromatography (HPLC) on a C18 column with a step gradient from 5% to 97% acetonitrile, as previously described (17, 18). We then tested each of the 12 subfractions for antifungal and antibacterial activity against *C. albicans* (CaCi-2) and *E. coli* (DH5α) with liquid growth inhibition assays (Fig. 2D and E). Only subfraction seven (F7) from WAC5038 displayed bioactivity against *C. albicans* in the presence of fluconazole (Fig. 2D). The lack of single-agent activity in any of the subfractions might be attributed to the dilution or loss of the active component(s) or a requirement of multiple components to observe bioactivity. On the other hand, subfractions four, five, and six (F4–F6) from WAC5287 displayed activity in the absence and presence of fluconazole (Fig. 2E). Unexpectedly, none of the twelve subfractions of WAC5038 had antibacterial activity against *E. coli* (Fig. 2E). This could be due to the loss or dilution of the active component(s) or a requirement of multiple components for bioactivity in WAC5038. WAC5287 did not display previous antibacterial activity (Fig. 2C); thus, the lack of activity seen in the subfractions was predicted (Fig. 2E). The UV/Vis absorbance spectra of fractions four, five, and six of WAC5287 had a characteristic polyene signature with three absorption peaks at 362, 382, and 402 nm (Fig. 2F), whereas fraction seven from WAC5038 did not have this signature. In support of the above, when testing WAC5287-conditioned medium against a *C. albicans* strain resistant to the polyene amphotericin B (ATCC 20095), a fourfold increase in resistance to WAC5287-conditioned medium was observed compared to a laboratory strain of *C. albicans* (SN95) (Fig. 2G). Surprisingly, the amphotericin B-resistant strain was fourfold more sensitive to WAC5038-conditioned medium than a wild-type strain (Fig. 2G). This suggests that WAC5038 produces a compound(s) that may be novel and active against drug-resistant *C. albicans* isolates, a focus for future studies.

In summary, this work outlines a pipeline to identify, isolate, and evaluate preliminary antimicrobial activity in microbes from both mammalian and environmental sources. Overall, this research enhances ongoing efforts to explore the mammalian-associated microbiota and environmental microbial reservoirs for natural products with antimicrobial activity.
